# CRISPR-Cas9 off-targeting assessment with nucleic acid duplex energy parameters

**DOI:** 10.1186/s13059-018-1534-x

**Published:** 2018-10-26

**Authors:** Ferhat Alkan, Anne Wenzel, Christian Anthon, Jakob Hull Havgaard, Jan Gorodkin

**Affiliations:** Center for Non-coding RNA in Technology and Health, Department of Veterinary and Animal Sciences, University of Copenhagen, Grønnegårdsvej 3, 1870 Frederiksberg, Denmark

**Keywords:** CRISPR-Cas9, Off-targets, Off-target scoring, Energy models, gRNA specificity, gRNA design

## Abstract

**Background:**

Recent experimental efforts of CRISPR-Cas9 systems have shown that off-target binding and cleavage are a concern for the system and that this is highly dependent on the selected guide RNA (gRNA) design. Computational predictions of off-targets have been proposed as an attractive and more feasible alternative to tedious experimental efforts. However, accurate scoring of the high number of putative off-targets plays a key role for the success of computational off-targeting assessment.

**Results:**

We present an approximate binding energy model for the Cas9–gRNA–DNA complex, which systematically combines the energy parameters obtained for RNA–RNA, DNA–DNA, and RNA–DNA duplexes. Based on this model, two novel off-target assessment methods for gRNA selection in CRISPR-Cas9 applications are introduced: CRISPRoff to assign confidence scores to predicted off-targets and CRISPRspec to measure the specificity of the gRNA. We benchmark the methods against current state-of-the-art methods and show that both are in better agreement with experimental results. Furthermore, we show significant evidence supporting the inverse relationship between the on-target cleavage efficiency and specificity of the system, in which introduced binding energies are key components.

**Conclusions:**

The impact of the binding energies provides a direction for further studies of off-targeting mechanisms. The performance of CRISPRoff and CRISPRspec enables more accurate off-target evaluation for gRNA selections, prior to any CRISPR-Cas9 genome-editing application. For given gRNA sequences or all potential gRNAs in a given target region, CRISPRoff-based off-target predictions and CRISPRspec-based specificity evaluations can be carried out through our webserver at https://rth.dk/resources/crispr/.

**Electronic supplementary material:**

The online version of this article (10.1186/s13059-018-1534-x) contains supplementary material, which is available to authorized users.

## Background

The CRISPR-Cas9 system, adapted from a bacterial defense mechanism, is a powerful genome-editing tool that recently revolutionized the field of biology, biotechnology, and medicine [1]. The system consists of the Cas9 protein and a guide RNA (gRNA) which together form a riboprotein complex (RNP) that can bind to gRNA-directed location on genomic DNA. Upon binding, Cas9 cleaves the DNA, making a double-stranded break which enables further DNA modifications on the site. As alternative Class II CRISPR systems, there exist variants of the Cas9 protein and other similar proteins with similar genome-editing potential, like Cpf1 [2], C2c1 [3], and C2c2 [4], but each comes with different targeting constraints and efficiency for the intended cleavage. Cas9 is the first CRISPR protein that has been adapted as a genome editing tool in eukaryotes [5] and has been successfully applied numerous times on many genomes such as yeast, human, and mouse. The CRISPR-Cas9 mechanism starts with the RNP complex recognizing the protospacer adjacent motif (PAM) in the target genome and then forming an RNA–DNA interaction duplex between the gRNA and the DNA on the opposite strand of the PAM upstream region [6–8]. However, gRNAs are mostly designed in a way that only the first 20 nt on the 5 ^′^ end are capable of forming this duplex. In the following, we by gRNA refer only to this 20-nt DNA binding region. Note that it is the only region that is changed when targeting different regions in the genome. When PAM recognition is supplemented with a stable gRNA–DNA duplex, Cas9 protein cleaves the DNA on both strands in a PAM-proximal region, usually 3 nt upstream from the PAM sequence. After this cleavage, DNA could be repaired with non-homologous end joining or homologous DNA repair, enabling insertion or deletion of DNA elements in specific regions. This special capability of the CRISPR-Cas9 system promises revolutionary innovations in the field of biology, biotechnology, and medicine, due to its efficiency and practicality as genome-editing tool [9].

For any CRISPR-Cas9 application, the very first step is to select a target region in the genome, which consequently determines the gRNA sequence to be used. Different gRNA selections have varying on-target cleavage efficiencies, and the underlying molecular mechanism is still not fully understood [10]. So far, several factors such as sequence context, stability of the gRNA binding, chromatin accessibility, and PAM sequence have been reported as influential factors, and several on-target efficiency prediction methods have been proposed to be able to predict the efficiency of intended cleavage (see [11] for a thorough discussion). Another design concern for gRNA selection has been the specificity of the intended cleavage. Even though the CRISPR-Cas9 mechanism is believed to be very specific to carry out the intended cleavage on genome, many studies reported that the Cas9 complex also binds to other unintended regions, called off-targets, and performs cleavage at these off-target sites as well [12–21]. It has been shown that off-target regions are gRNA-specific and that they usually are highly homologous to the intended on-target region. When compared with on-target sites, reported off-target regions generally have up to six mismatches and off-targets with fewer mismatches tend to have more prominent binding and cleavage. Several tools have been developed to find potential off-target regions for given gRNA sequences and they mainly focus on finding off-targets in the genome of interest, allowing up to a certain number of mismatches [22]. However, initial analyses on experimentally reported off-targets showed that the type of mismatch and its distance from the PAM sequence also have significant importance. This information enabled the development of several off-target scoring methods and helped researchers to select their gRNAs with information on their off-targeting potential (see [11, 22] for a thorough discussion).

In this study, we developed novel off-target and specificity scoring methods distinctively by using a biophysical interaction model for Cas9–gRNA–DNA binding. There have been recent efforts to develop biophysical models for Cas9 binding [23–25]; however, none of the models actively made use of the free energy and enthalpy change parameters estimated for nucleic acid duplexes from experimental measurements [26–31]. These duplex-specific parameters enable computation of the free energy of nucleic acid duplexes, and they have been proven to be quite useful for intra- and inter-molecular interaction prediction of RNA molecules [32]. The base pair-specific nature of nucleic acid duplex energy models can potentially explain why some mismatches are more common within reported off-target regions and they can be quite helpful to accurately compute the stability of any Cas9 binding. Thorough details about how we obtain these parameters and make use of them within our scoring methods are given in the “Methods” section.

## Results

To assess the off-targeting potential of gRNA selections in CRISPR-Cas9 applications, we developed two novel scoring methods, CRISPRoff and CRISPRspec. The former calculates an off-target score based on our energy model that approximates the free energy of any gRNA–DNA binding, and the latter provides a specificity score by making use of free energies computed for all possible on- and off-target bindings.

**Fig. 1:**
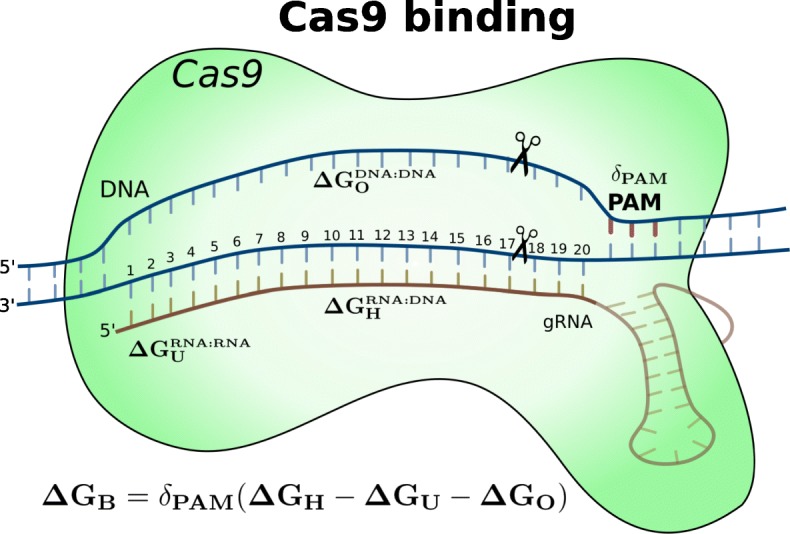
Players of the energy model that determines the approximate free energy (*ΔG*_*B*_) of any Cas9–gRNA–DNA binding. In this model, that is posterior to the Cas9–gRNA binding, there are four main contributions to the overall free energy. The first contribution is *ΔG*_*H*_ for the gRNA–DNA hybridization, computed with RNA–DNA duplex energy parameters and weighted by a position-wise estimate of the Cas9 influence in the binding. The second contribution is the *ΔG*_*O*_ penalty to open the DNA–DNA duplex in the target region and it is computed with DNA–DNA duplex energy parameters. The third contribution is the *ΔG*_*U*_ penalty that is the free energy of the gRNA (first 20 nt) folding. This is computed with RNAfold program which incorporates RNA–RNA duplex energy parameters [32]. The fourth contribution is the correction factor *δ*_*PAM*_ that is determined by the PAM sequence of the target

Our approximate free energy model is depicted in Fig. 1. It includes calculating a position-weighted binding energy between gRNA and the (off-)target DNA (*ΔG*_*H*_), the free energy of the DNA duplex (*ΔG*_*O*_), the folding energy of the gRNA only (*ΔG*_*U*_), and a correcting factor (*δ*_*PAM*_) corresponding to the type of PAM sequence. As full energy models are not available, we have made approximate models. The details of the model, parameters, and approximation are described in the “Methods” section. In brief, the CRISPRoff score is a score for a specific individual off-target binding and is equal to the negative of *ΔG*_*B*_ shown in the figure and Eq. (4) (“Methods”). The CRISPRspec score is the ratio of the Boltzmann-weighted energies of all possible but the binding energy *ΔG*_*B*_ over the on-target region, to the Boltzmann-weighted energies of all possible bindings including the on-target binding energy as listed in Eq. (5) (“Methods”). Hence, the CRISPRoff score can be considered as a confidence score assigned to predicted off-target sites of a gRNA and CRISPRspec score represents the specificity of this gRNA, or conversely its overall off-targeting potential.

In the following, we present our evaluation results for both methods, followed by our findings on the relationship between on-target cleavage efficiency and specificity of different gRNA selections.

### Evaluation of off-target scoring methods

There exist a few methods in the literature that assign confidence scores to predicted off-target sites and we benchmarked our novel method CRISPRoff with six of them, CCTop [33], CFD [34], Cropit [35], Elevation (Elevation score) [36], MIT [11, 16], and VfoldCAS [24]. We benchmarked these methods under three different evaluation settings. First, we compared the performance of the methods with receiver operating characteristic (ROC) analysis using the recently published Haeussler benchmark dataset that evaluated the performance of off-target scoring algorithms in a similar sense [11]. This dataset contains 650 off-target sequences reported for 31 different gRNAs and it is a collection of experimentally supported off-targeting data from 8 different studies [14–21]. Haeussler et al. originally used only a small portion of this data for their evaluation, limiting the ROC analysis to off-target predictions with up to four mismatches, excluding two of the gRNAs which had the highest number of off-targets and two of the assays that use targeted sequencing [14, 16], due to their low sensitivity [11]. In our analysis, assays that are classified as low-sensitivity by Haeussler et al. are also excluded; however, for a more comprehensive evaluation of off-target scoring methods, the two gRNAs with highest number of reported off-targets are included. We assume that the more off-targeting data taken into account, regardless of the volume of off-targets reported for one gRNA, the more comprehensive the performance assessment of off-target scoring methods becomes. We allow up to six mismatches in off-target predictions to include all experimentally supported off-targets (true positives) within the ROC analysis. Note that off-target predictions of the gRNAs in this dataset were also obtained from the benchmark dataset itself. Within the final ROC analysis set, we had 605 true positive (experimentally-supported) off-targets (with PAM sequences of NGG, NAG, or NGA) reported for 26 unique gRNAs, where total number of off-target predictions with up to six mismatches was equal to 1167036.

**Fig. 2:**
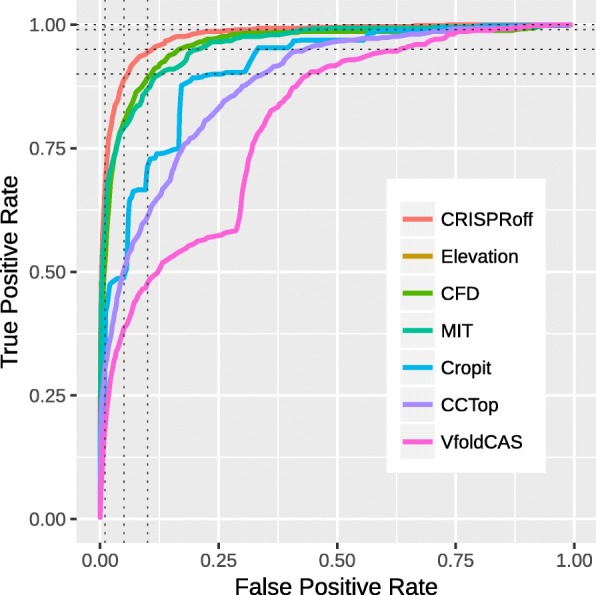
Receiver operating characteristic (ROC) analysis of off-target scoring methods when benchmarked with the Haeussler dataset [11], allowing up to six mismatches, and NGG, NAG, and NGA PAM sequences for off-targeting. ROC curves for CFD and Elevation methods largely overlap and CRISPRoff shows the best performance with the largest area under its ROC curve. FPR and TPR values of the methods at specific points, indicated by dashed lines, are given in Table 1

In Fig. 2, we present our ROC analysis where the true positive rate (TPR) and its corresponding false positive rate (FPR) are reported at method-specific varying thresholds. One can readily see that energy-based off-target score CRISPRoff performs better than all other methods with its higher area under the curve. For completeness, the precision-recall (PR) curve of this analysis is given in Additional file 1: Figure S1, where TPR and corresponding positive predictive values (PPV) are reported for each method. The PR curve also supports that CRISPRoff is the top performer with its highest area under the curve. A summary of the statistics from the ROC analysis is given in Table 1. In addition to higher area under ROC and PR curves, it is very clear that CRISPRoff outperforms all other methods with lower FPR and higher TPR values at given fixed TPR and FPR values, respectively. For example, when CRISPRoff score reaches 0.9 TPR, its FPR is 0.06 which is almost two times better than the closest competitors (CFD and Elevation). Note that, at this fixed TPR, the performance gain of CRISPRoff over these methods actually corresponds to > 58k fewer FPs in off-target predictions.

**Table 1:** Area under ROC (TPR vs. FPR) and precision-recall (PPV vs. TPR) curves for off-target scoring methods when benchmarked with the Haeussler dataset [11], allowing up to six mismatches, and NGG, NAG, and NGA PAM sequences for off-targeting

		Off-target scoring method						
*Area*		CRISPRoff	Elevation	CFD	MIT	Cropit	CCTop	VfoldCAS
ROC		**.98**	.96	.96	.96	.91	.88	.80
PR		**.18**	.08	.08	.12	.05	.06	.01
TPR								
.9	FPR	**.06**	.11	.11	.13	.27	.34	.44
.95		**.11**	.17	.17	.21	.33	.44	.63
.99		**.32**	.88	.88	.44	.71	.74	.84
1		**.73**	.97	.97	.96	.99	.91	.96
FPR								
.01	TPR	**.67**	.52	.52	.59	.36	.31	.18
.05		**.89**	.80	.80	.79	.49	.50	.39
.1		**.94**	.89	.89	.87	.71	.61	.48

Corresponding TPR and FPR performance of the methods are also given for some fixed FPR and TPR values. Best performances are given in bold

**Fig. 3:**
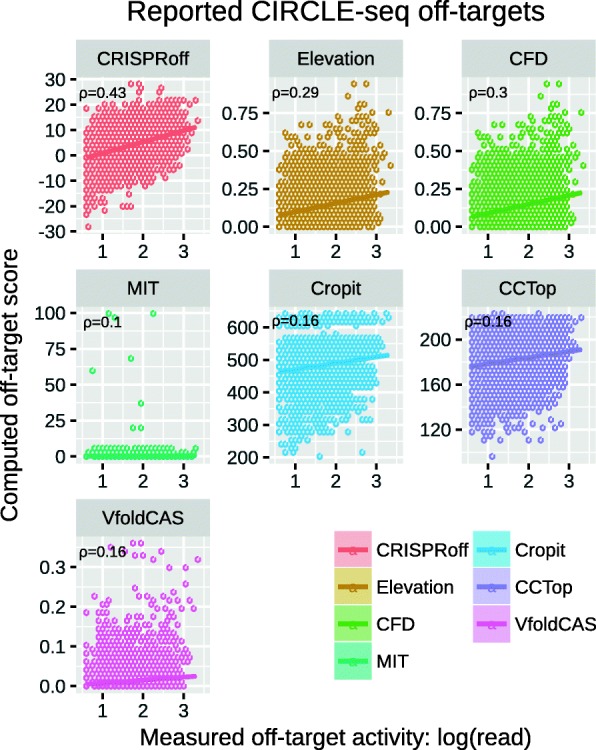
Method-specific off-target score vs. off-target activity scatterplots (hexagonal binned) with all reported off-targets from CIRCLE-seq dataset. Measured off-target activity, given on the *x*-axis, corresponds to the logarithm of read counts reported for that specific off-target region. Fitted lines are shown together with the Pearson correlation coefficient between *x-* and *y*-axis variables in the top left corner of each subplot

In our second benchmark setting, we investigated how well different off-target scoring methods agree with the cleavage efficiency of the experimentally reported off-target regions. In these analyses, the recently published CIRCLE-seq [37] and SITE-seq [38] experimental datasets were used. In CIRCLE-seq dataset, off-targets are reported in 19 experiments using 11 different gRNAs, whereas this is done for 8 gRNAs at 5 different concentrations within the SITE-seq dataset. Both methodologies detect the gRNA-specific off-targets on a genome-wide level and they provide read counts for cleaved off-target regions in the human genome, representing their cleavage efficiency. In the CIRCLE-seq dataset, some gRNAs are tested multiple times in different cell lines and it is shown that off-targeting is more gRNA-specific than cell-line-specific. In the SITE-seq dataset, experiments at different concentrations show that as the concentration of Cas9 complex increases, the off-targeting effects become more prominent. Within the evaluation, we first made use of the CIRCLE-seq dataset excluding one experiment where the gRNA did not have any perfect complementary target in the human genome (hg38). Each subplot in Fig. 3 indicates the performance of different off-target scoring methods on CIRCLE-seq dataset. In these plots, positive correlation between off-target scores and cleavage efficiencies hints to better performance and it is clear that CRISPRoff score is in best agreement with measured off-target activity over all CIRCLE-seq reported off-targets under consideration. This is supported by the CRISPRoff score having the highest Pearson correlation coefficient (*ρ*), which is given in the top-left corner of each plot. Closest to this are the CFD and Elevation scores, which is also in agreement with the ROC analysis above. The analysis with the SITE-seq dataset is however more blurry and does not support this as significantly as the CIRCLE-seq dataset. The correlation between off-target scores and their cleavage efficiency reported by the SITE-seq method is very weak for all methods (see Additional file 1: Figure S2).

**Fig. 4:**
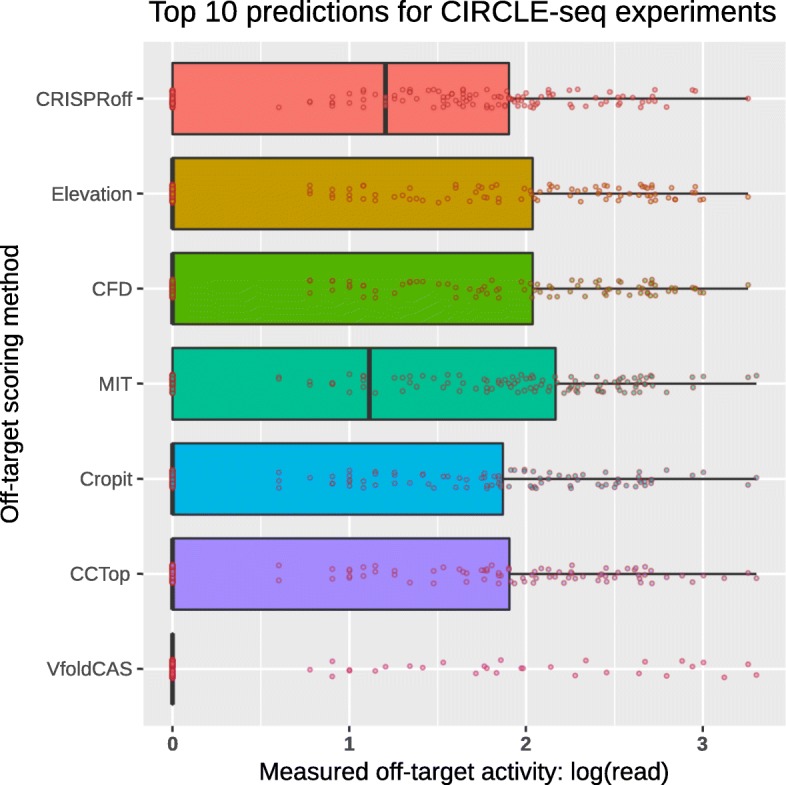
CIRCLE-seq measured off-target activity distributions of method-specific top predictions (180 in total, top 10 for all 18 experiments). Distributions are given separately for each method in box plot format combined with log(read) values for each off-target prediction as dot plots. Value 0 in *x*-axis corresponds to no experimental support for that off-target prediction

In our third benchmark, we evaluated the off-target scoring methods with their accuracy in their top predictions. For every experiment in the CIRCLE-seq dataset, we used the RIsearch2 [39] program to obtain the list of potential off-target sites, up to six mismatches in human (hg38) genome (see the “Methods” section for details), and filtered them with PAM sequences of NGG, NAG, or NGA. These were then ranked by each of the off-target scoring method. Focusing solely on the top 10 off-target predictions of each method for all 18 experiments (180 predictions in total), the distribution of measured off-target activities was compared in Fig. 4. One can see that top off-targets identified with the CRISPRoff and MIT methods have the lowest number of false positives since more than half of their top predictions have cleavage support from the CIRCLE-seq experimental dataset. The median measured off-target activity values of the top off-targets from the CFD, Elevation, Cropit, CCTop, and VfoldCAS methods are equal to 0, indicating more than half of their top predictions have no experimental support. The median values of ∼ 1.0 for CRISPRoff and MIT methods, suggest similar outperformance of all the other methods for both of these. The corresponding analysis on the SITE-seq data set is presented in Additional file 1: Figure S3. However, in this analysis, the methods show closer performances, except the poor performance of VfoldCAS, Elevation, and CFD.

All in all, our findings from all the benchmarks presented above suggest that the CRISPRoff method consistently outperforms the other off-target scoring methods when assigning confidence scores to predicted off-target regions. This is supported by its stronger agreement with experimentally reported off-targets, especially in the CIRCLE-seq dataset, not only in classification but also at cleavage efficiency correlation level.

### Evaluation of gRNA specificity scores

Apart from assigning confidence scores to the off-target predictions of a gRNA, another challenge for Cas9 off-targeting assessment is to assign specificity scores to different gRNA selections. To the best of our knowledge, there exist two methods in the literature that can perform this task, namely the MIT [16] and Elevation (Elevation-aggregate) [36] methods. With this study, we propose a novel approach, CRISPRspec, to measure the specificity of any given gRNA targeting a selected genome. For more accurate evaluation of the CRISPRspec, Elevation, and MIT methods, we use two versions of the MIT specificity score, indicated as MIT and MIT*. The former MIT score is computed by the CRISPOR webserver [11] where off-target space is limited with four mismatches as default and the recommended threshold is 50 to bin the gRNAs into high or low specificity groups. The latter MIT score, MIT*, is computed using the code from the Haeussler benchmarking study [11] with a different off-target prediction set given as input, that is the set used for computing the CRISPRspec score. For any given gRNA, this set is generated by using RIsearch2 [39], allowing up to six mismatches between gRNAs and their targets in the human genome (hg38), followed by post-filtering with the PAM sequences of NGG, NAG, and NGA. On the other hand, Elevation score is computed using its own off-target prediction set which also allows up to six mismatches and same PAM sequences.

Performances of the CRISPRspec, Elevation, MIT, and MIT* scores are compared using the SITE-seq and CIRCLE-seq datasets. However, evaluation with the SITE-seq dataset is our primary focus since all experiments from this dataset are performed in the same type of cell line. We assume that in this way, we can minimize the potential evaluation error that is caused by different chromatin accessibility patterns of the cells, a parameter that is not taken into account in all methodologies. Besides, the SITE-seq dataset enables assessing the accuracy of specificity scores at different concentrations.

**Fig. 5:**
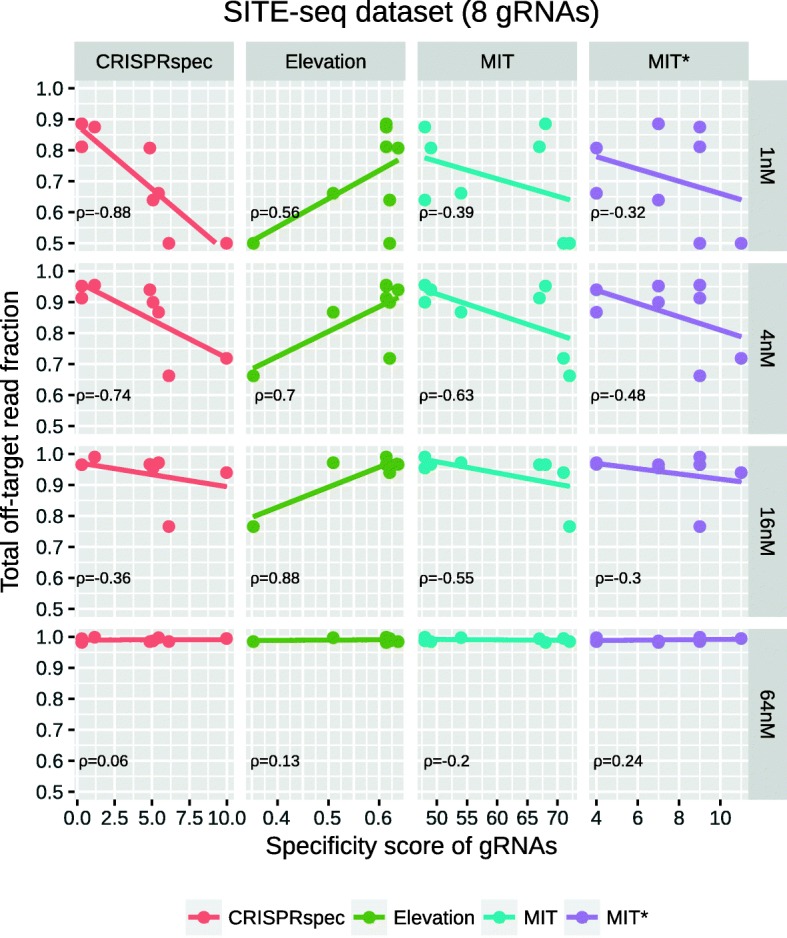
Total off-target activity reported by the SITE-seq experiments vs. method-specific specificity scores for eight unique gRNAs. For each gRNA, the CRISPRspec and MIT* scores have been computed with the same set of off-target predictions allowing up to six mismatches, whereas Elevation scores are based on its own prediction set (up to six mismatches) and MIT score has been computed with CRISPOR tool [11] allowing up to four mismatches in off-target predictions by default. Results regarding to different concentration levels in the SITE-seq dataset are given separately at each row. Fitted lines are shown together with the Pearson correlation coefficient between *x-* and *y*-axis variables in the bottom left corner of each subplot

In our evaluation with any of the datasets, we first compute the specificity of gRNAs in that group with all three methods and analyze its agreement with the experimentally measured specificity. The latter is represented by the fraction of off-target read counts within the total read count reported for that gRNA in that dataset. Evaluation results with the SITE-seq dataset at four different concentrations are shown in Fig. 5 where the *x*-axis indicates the predicted specificities and the *y*-axis shows the experimentally measured specificities of the gRNAs. It is expected that gRNAs with higher specificity have a lower fraction of off-target read counts, and therefore, stronger negative correlation between the two measures hints to better performance for that method. Focusing on the first row in Fig. 5, the lowest concentration experiments in the SITE-seq dataset, one can see that CRISPRspec specificity score is in best agreement with experimental results due to lower off-targeting activity for highly specific gRNAs and higher off-targeting activity for the low specificity ones. However, agreement with the experimentally measured specificity is much weaker for MIT and MIT* scores and weakest for Elevation method. For the results in the other concentration levels (rows 2–4 in Fig. 5), it is clear that the experimental evidence for specificity differences between gRNAs disappears at higher concentrations so as the agreement between experimental and predicted specificity measures.

The results concerning the CIRCLE-seq dataset are given in Additional file 1: Figure S4, which also suggests that CRISPRspec is the top performer (*ρ*=−0.72) when compared to MIT (*ρ*=−0.49), MIT* (*ρ*=−0.05) and Elevation (*ρ*=0.20) methods.

### Specificity and on-target efficiency interplay for gRNAs

On-target cleavage efficiency of a gRNA is influenced by various factors, from gRNA/target sequence context to genomic location of the target, and there are several tools with varying performance that take these factors into account for efficiency prediction of the selected gRNA [11]. However, predicted specificity measure of different gRNA selections is usually not part of on-target efficiency scoring schemes since this relationship is believed to be insignificant. Here, we reanalyze this potential interplay using both numerical (specificity measure) and experimental (cleavage efficiency) data for two groups of gRNAs, Doench2015 [40] (881 gRNAs) and Wang2015 [41] (2921 gRNAs). Firstly, the CRISPRspec and MIT* specificity score of these gRNAs are computed and they are assigned into low, medium, and high specificity groups within the respective data sets. The binning thresholds for CRISPRspec and MIT* scores are selected in a way that they would create three equal-sized specificity groups for 57980 unique gRNAs that target 16322 different genes in the human genome [42]. Secondly, we compare the distribution of experimentally measured on-target cleavage efficiencies of the gRNAs that are binned into different specificity groups.

**Fig. 6:**
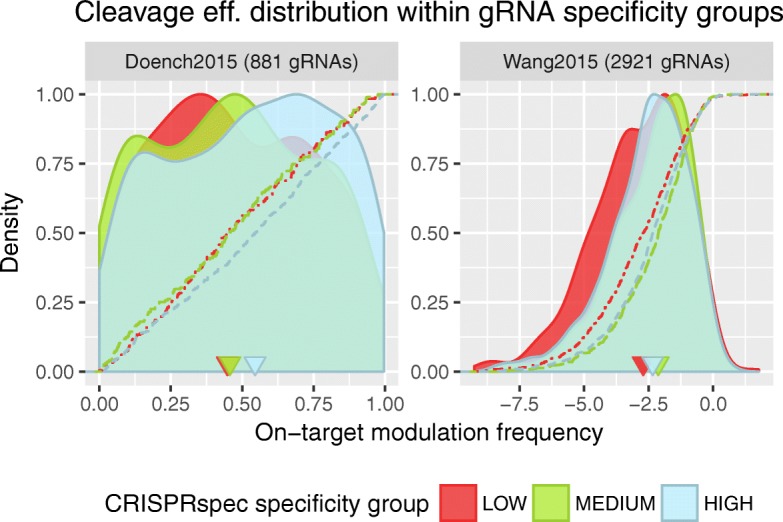
On-target modulation frequency distribution of gRNAs that are binned into low, medium, and high specificity groups using CRISPRspec method. Distributions are given as kernel density estimates (filled curves) together with the cumulative distribution function (dashed lines) of on-target modulation frequencies for each specificity group, separately for each dataset. Given modulation frequencies represents the cleavage efficiency of the intended on-target and are dataset specific. Triangles on the *x*-axis indicates the median values

In Fig. 6, one can see that efficiency distribution of low and high specificity groups are skewed towards opposite ends, indicating that low specificity gRNAs are more likely to have less on-target efficiency and highly specific gRNAs are more likely to be more potent for their intended cleavage. This is supported by pairwise Kolmogorov–Smirnov (K–S) tests within each dataset, indicating significant differences (*p*value < 0.05) between the on-target modulation frequency distribution of gRNAs from different specificity groups (except the test between low and medium specificity group for Doench2015 dataset). When using the MIT* score instead of the CRISPRspec score for the specificity grouping of gRNAs, this interplay, with higher confidence on Doench2015 dataset (lower *p* values in K–S tests), is still supported. However, this is not the case for MIT* score with the Wang2015 dataset (see Additional file 1: Figure S5). Out of the three pairwise K–S tests within the Wang2015 dataset, K–S tests for low-vs-medium and medium-vs-high specificity groups yield to *p* values larger than 0.05, whereas the low-vs-high K–S test yields a *p* value equal to 0.045. Failure of these two K–S tests with MIT* scores in Wang2015 dataset could also be interpreted as a sign of CRISPRspec outperforming MIT* score.

Over all, these findings provide a considerable support for the parallel relationship between the specificity and the on-target efficiency of gRNAs and suggests that off-target volume of gRNAs might have negative impact on the efficiency of their on-target cleavage. Therefore, integration of the CRISPRspec specificity measure to gRNA efficiency prediction tools can potentially improve their performances.

## Discussion

Prior to any CRISPR-Cas9 genome-editing application, computational on- or off-targeting assessment of gRNAs is a crucial step to be able to select the most efficient gRNAs with minimum off-targeting effect. With this study, we proposed two novel methods for computational off-targeting assessment, CRISPRoff and CRISPRspec. The CRISPRoff off-targeting score can be interpreted as a confidence score that is assigned to the predicted off-targets of a gRNA and the CRISPRspec specificity score is a measure for the specificity/off-targeting potential of a gRNA. Both of the methods are based on an approximate energy model for Cas9–gRNA–DNA binding which is another novel outcome of this study. The model proposed here uses the nucleic acid duplex energy parameters for free energy computation, taking all RNA–RNA, RNA–DNA, and DNA–DNA interactions into account.

In our benchmark analysis with the latest experimental off-target screening datasets, we showed that CRISPRoff and CRISPRspec scores are more accurate than other available off-target and specificity scoring methods, making them the new state-of-the-art methods for computational off-targeting assessment of CRISPR-Cas9 gRNAs. Their strong agreement with the experimental off-target screens shows that they hold great potential to serve as gRNA design criteria prior to all Cas9 genome-editing applications. For the selection of gRNAs, CRISPRoff score can help with accurate ranking of predicted off-target regions, whereby gRNAs with high confidence off-targets on important regions of the target genome could be discarded in the first place. In addition, when the volume of off-targeting is a bigger concern than the individual off-target regions, CRISPRspec specificity score can help with pre-filtering of the gRNA selections based on their measured specificity on the target genome. Due to the potential interplay we have shown between the specificity and on-target cleavage efficiency of gRNA selections, selecting highly specific gRNAs can also increase the chances of successful on-target cleavage for Cas9 applications. As a result, these two novel methods, CRISPRoff and CRISPRspec, provide more accurate off-targeting assessment of gRNA selections and can help researchers to use the CRISPR-Cas9 system with higher efficiency and security.

All benchmarks given in this study are focused on the human genome, simply due to the number of datasets available for human. However, more off-targeting data is becoming available for other organisms as well and we consider the benchmarks on other genomes as part of our future work. The starting point for such benchmarks could be the Anderson2018 dataset, where a few thousand off-target regions are tested for over hundred gRNAs in mouse and rat genomes [43].

As more future work, our free energy-based approach applied here could provide further understanding about the details of the Cas9 binding and cleavage machinery, whether it is on- or off-target. Moreover, our analysis on the specificity-efficiency interplay suggests that predicted specificity measure of gRNAs, like CRISPRspec, could be incorporated into gRNA design tools and this might enhance the efficiency prediction for gRNA selections.

The methods proposed here solely focus on CRISPR-Cas9 system; however, they can easily be adapted to other CRISPR proteins as well. This would require minor reformulations in the approximate energy model and some of the Cas9-related weights would need to be retrained for the CRISPR protein of interest. These weights could be trained using protein-specific experimental off-targeting and/or biochemical profiling data, as we did here using a biochemical profiling dataset [25] for Cas9 off-target interactions (see “Methods” section). Additionally, our partition function-based approach can incorporate the abundance information of targets as well. This also holds great potential to be applied to off-targeting assessment of RNA-targeting CRISPR proteins, like Cas13 [44]. This approach has been successfully applied to siRNA off-target predictions before [39] and transforming this approach into CRISPR applications is part of the future work.

## Conclusions

The performance of the CRISPRoff off-target scoring method and the CRISPRspec gRNA specificity measure not only enables more accurate off-target evaluation of gRNA selections. They imply that the binding energies have a substantial impact on off-targeting mechanisms, which also provides a direction for further studies. Prior to any CRISPR-Cas9 genome-editing application, the CRISPRoff-based off-target predictions and the CRISPRspec-based specificity evaluations can be carried out through our webserver at https://rth.dk/resources/crispr/.

## Methods

### Approximate free energy model for Cas9 binding

Our observations, along with recent studies [23], support that the binding affinity of the Cas9–gRNA–DNA complex controls not only the occupancy of the target DNA but also influences the cleavage rate of it. Denoting any Cas9 complex binding with *B*[*g*,*t*] and its free energy with *ΔG*_*B*[*g*,*t*]_, for a gRNA *g* and a target DNA *t*, our approximate free energy computation consists of four components: (i) the free energy contribution of gRNA–DNA hybridization (*ΔG*_*H*_[*g*,*t*]), (ii) the energy penalty for unfolding the gRNA itself (*ΔG*_*U*_[*g*]), (iii) another penalty for opening (melting) the double-stranded DNA (*ΔG*_*O*_[*t*]), and (iv) a final energy correction *δ*_*PAM*[*t*]_ based on the PAM sequence of the target *t*. These components make up the full energy model illustrated in Fig. 1, and the equation in the figure summarizes the free energy approximation of any binding site *t* for a given gRNA *g*.

To be able to compute all the *ΔG* free energy contributors, we made use of the Turner [26] and SantaLucia [27] nearest neighbor energy models for RNA–RNA and DNA–DNA duplexes, respectively. Note that we also used the parameters from the Allawi energy model [30] to complement some of the missing parameters of the SantaLucia model for DNA–DNA duplexes, e.g., G-T mismatches. A summary of these models can be found in the Additional file 1: Section 2. For the RNA–DNA duplex energy model, we primarily used the Sugimoto [28, 29] and Watkins [31] energy models to obtain the free energy parameters for stacked base pairs and some specific single mismatches. Due to the lack of the full energy parameters [23], we simply averaged the DNA–DNA and RNA–RNA parameters to complete the missing parameters of this model. The same approach was also used in the ViennaRNA package [45]. Our resulting nearest neighbor energy models for all three duplexes include base pair stacking energy contributions, penalties for mismatches within internal loops, and specific energy contributions of the internal loops at varying lengths. Further details about the nucleic acid duplex parameters are given in Additional file 1: Section 2. Note that, within the current models, we ignore the energy parameters for bulges since we only score mismatched off-target predictions. This is a common limitation for all off-target scoring methods; however, it is not a concern since bulged off-targets have been rarely reported at very low cleavage rates.

Each of the four contributions to our energy model mentioned above are determined as follows.

(i) *ΔG*_*H*_*[g,t]:* This contribution is obtained by summing up the estimated RNA–DNA interaction parameters. However, due to the influence of the Cas9 protein, we weight these for each position *i* in the interaction (1≤*i*≤19), by a factor *Γ*_*Cas*9_[*i*] explained below. Thus we compute *ΔG*_*H*_[*g*,*t*] as

1 $$\begin{array}{@{}rcl@{}} {}\Delta G_{H}[g,t] = \sum\limits_{i=1}^{19} \Gamma_{{Cas9}}[i] \times \Delta G_{g[i,i+1]:t[i,i+1]}^{{RNA:DNA}}, \end{array} $$

where $\Delta G_{g[i,i+1]:t[i,i+1]}^{{RNA:DNA}}$ is the estimated free energy contribution of the stacked match (or mismatch) between the gRNA and the target DNA sequence at position *i*. When Watson–Crick base pair matches are stacked on each other, the free energy contribution of position *i* depends only on the (*i*)th and (*i*+1)th bases (*g*[*i*,*i*+1] and *t*[*i*,*i*+1]), where the order of *i* is from 5 ^′^ to 3 ^′^ end of the gRNA and the other way around (3 ^′^ to 5 ^′^) for the DNA (see Fig. 1 and Additional file 1: Figure S6). However, interactions formed between gRNAs and off-targets usually contain mismatches and they create interior loops in the RNA–DNA duplex. As explained above, in regions with stacked Watson–Crick base pairs, every stacking pair contributes individually at each position; however, for interior loops, we compute the overall energy of the interior loop and divide it equally to all positions forming the loop as positional contributions. In Additional file 1: Figure S6, we provide an example gRNA–DNA binding and explain how to compute its positional free energy contributions in Additional file 1: Section 2.1.3.

The influence of the Cas9 protein is modeled heuristically by generating positional weights, *Γ*_*Cas*9_[*i*], for the energy contribution at each position *i* of the gRNA–DNA binding (1≤*i*≤19). The base pair stability at different positions of this binding might have different impacts due to the conformation of Cas9 protein and this impact can be trained on biochemical profiling datasets that can measure the kinetics of different gRNA–target bindings. Here, we used a recently published biochemical profiling dataset for Cas9 off-target bindings [25], where association and dissociation rate of nuclease-dead dCas9 interactions are measured with a massively parallel method. Our estimation of *Γ*_*Cas*9_[*i*] parameters are done as follows: For one specific gRNA, denoted with $\hat {g}$, this dataset provides initial association rates across a range of potential off-target sequences. We denote this off-target set with *O*, every individual off-target with $\hat {o}_{n}$ and its association rate with $\tilde {a}_{n}$, where 1≤*n*≤|*O*|. First, for every off-target $\hat {o}_{n}$, we compute the energy contribution of 19 base pair stackings individually, between the gRNA and that specific off-target. Then, for each position *i* in the stack, we calculate the *W*_*i*_ position-specific weighted sum of the energy contributions over all off-targets, where the weight is the association rate $\tilde {a}_{n}$ for every $\hat {o}_{n}$. Finally, to transform these *W*_*i*_ weighted sums into *Γ*_*Cas*9_[*i*] positional weights, where the lowest positional weight is desired to be 1 with no large deviations from this value, we normalize them with the minimum sum, take its logarithm, and sum it with 1. This computation is formulated in Eq. (2) below and our final set of values have been computed as *Γ*_*Cas*9_= {1.80, 1.96, 1.90, 2.13, 1.38, 1.46, 1.00, 1.39, 1.51, 1.98, 1.88, 1.72, 2.02, 1.93, 2.08, 1.94, 2.15, 2.04, 2.25}. The obtained values show the importance of the PAM-proximal region with consistently higher weights.

2 $$\begin{array}{*{20}l}  \Gamma_{{Cas9}}[\!i] &= \log_{10}\left({W}_{i}/ \min\limits_{W_{1}\ldots W_{19}}\right)+1 \\ with \, {W}_{i} &= \sum\limits_{n=1}^{|{O}|} \tilde{a}_{n} \times \Delta G_{\hat{g}[i,i+1]:\hat{o}_{n}[i,i+1]}^{{RNA:DNA}}  \end{array} $$

(ii) *ΔG*_*U*_*[g]:* For this we use the RNAfold program [32] with gRNA sequence that binds to the target DNA given as input (first 20 nt), and obtain the free energy of predicted MFE structure. Note that for some gRNA sequences, this value is equal to zero due to lack of predicted folded structure.

(iii) *ΔG*_*O*_*[t]:* Similar to the RNA-DNA interaction, this is obtained by summing up the estimated DNA-DNA interaction parameters:

3 $$\begin{array}{@{}rcl@{}}  \Delta G_{O}[t] = \sum\limits_{i=1}^{19} \Delta G_{t^{\prime}[i,i+1]:t[i,i+1]}^{{DNA:DNA}}, \end{array} $$

where we note that $\phantom {\dot {i}\!}\Delta G_{t^{\prime }[i,i+1]:t[i,i+1]}^{{DNA:DNA}}$ represents the duplex-specific nearest neighbor energy models as explained above. Since the DNA–DNA duplex (target *t* and its complement *t*^′^) at the target site is always perfect-complimentary, we only use the stacking energies of Watson–Crick pairs from DNA–DNA duplex energy parameters, for this computation. As can be seen from the equation above, every stacking position (*i*,*i*+1) contributes individually to the overall free energy where the direction for *i* is from 3 ^′^ to 5 ^′^ end for target DNA *t* and the other way around (3 ^′^ to 5 ^′^) for its complement *t*^′^. We provide the stack-specific energy parameters, based on SantaLucia [27] and Allawi [30] energy models, in Additional file 1: Table S2.

(iv) *δ*_*PAM[t]*_: The PAM sequence in the target DNA region is assumed to be responsible for the initial Cas9 recognition but the stability of the Cas9–gRNA–DNA complex is maintained through the RNA–DNA binding. Therefore, we decided to introduce the effect of PAM sequence to the overall binding stability with a parameter *δ*_*PAM*_ that influence the computed overall binding free energy. Values for *δ*_*PAM*_ have been selected arbitrarily for Cas9, as 1.0, 0.9, and 0.8 for the PAM sequences of NGG, NAG, and NGA, respectively. These values solely reflect our observations in the literature for experimentally validated off-targets of Cas9.

### CRISPRoff and CRISPRspec scores

For a given gRNA *g* and off-target *t*_*off*_, CRISPRoff score is simply equal to the estimated free energy contribution of the off-target binding *ΔG*_*B*_[*g*,*t*_*off*_]. However, CRISPRspec score computation is more comprehensive since we use a partition function approach from statistical thermodynamics to model the ensemble of all potential interactions. This model has already been proposed for CRISPR applications by Farasat and Salis [23], and it has been successfully applied to siRNA off-targeting assessment before [39]. Through the partition function, we simply compute the summed probability of all potential off-target interactions and propose its negative logarithm as our CRISPRspec specificity score. For a given gRNA *g*, denoting its set of target predictions with $\mathcal {T}_{g}$ including the intended target *t*_*on*_, and the thermodynamic constant with *β*, below equations summarize how CRISPRoff and CRISPRspec scores are computed.

4 $$\begin{array}{*{20}l}  {}\text{\texttt{CRISPRoff}} &[g,t_{{off}}]\\ =-&\Delta G_{B}[g,t_{{off}}] \\ = -&\delta_{{PAM}}\left(\Delta G_{H}[g,t_{{off}}]-\Delta G_{O}[t_{{off}}]\right. \!\!\!\left.-\Delta G_{U}[g]\right) \end{array} $$

5 $$\begin{array}{@{}rcl@{}}  {}{\text{\texttt{CRISPRspec}}[g,\mathcal{T}_{g}]\,=\,-\!\log_{10}\!\left(\!\frac{\sum\limits_{\forall t \in \mathcal{T}_{g} \setminus \{t_{{on}}\}} e^{-\beta \Delta G_{B}[g,t]}}{\sum\limits_{\forall t \in \mathcal{T}_{g}} e^{-\beta \Delta G_{B}[g,t]}}\!\right) } \end{array} $$

### Other off-target and specificity scoring methods

To compute the other off-target scores that are benchmarked here except the VfoldCAS and Elevation scores (see below), we simply made use of the code implemented in the Haeussler benchmarking study [11]. According to this study, some of these codes were taken from original sources but some were simply implemented by Haeussler et al. according to corresponding papers. For more information about this source code, please see the corresponding benchmark paper [11]. For the VfoldCAS score computation, we used its webserver [24] by uploading the gRNA and off-target sequences when needed.

Elevation scores have been computed using the stand-alone version of the tool (v3.3) that is downloaded through its github page. For any gRNA, both Elevation score (off-targeting) and Elevation-aggregate (specificity) scores have been computed using its own set of off-target predictions since it does not accept user-defined off-target sequences. However, when running the tool, we did not limit the number of off-target predictions and allowed up to six mismatches with NGG, NGA, and NAG PAM sequences (by passing the following arguments: –forcePamListNGG,NAG,NGA-t 6–matchSiteCutoff 0). When benchmarking the off-targeting scores, computed Elevation scores were parsed from the output files of the tool and assigned to corresponding off-target sequences. Note that off-target sequences that we could not compute an Elevation score for have been excluded from the analysis.

Lastly, to compute the original MIT specificity score, we ran the stand-alone version of the CRISPOR tool (v4.2) [11], allowing up to four mismatches between gRNAs and potential off-targets as it is the default option. However, since our CRISPRspec score was computed with our in-house predictions, we computed the updated MIT* score using the source code provided by the benchmark study [11].

### Benchmarking datasets

For evaluation purposes, we used three different off-targeting datasets. The dataset used for ROC analysis is taken from the benchmarking study [11] through its GIT repository, accessed in June 2017. The downloaded data includes 31 gRNA sequences, 718 reported off-targets, and all off-target predictions with up to four, five, or six mismatches have been generated using the provided code. Note that, as default, NGG, NAG, and NGA were all allowed as PAM sequences in off-target predictions given here. The area under ROC and PR curves were computed using the PRROC [46] package in R environment.

The other two datasets used for benchmarking are the CIRCLE-seq and SITE-seq datasets. For each of the datasets, we downloaded the gRNA sequences (11 in CIRCLE-seq, 8 in SITE-seq) and the reported off-targets (5563 in CIRCLE-seq, 5847 in SITE-seq), along with their read counts from the corresponding supplementary material of the papers. For the off-target predictions of these gRNAs in human genome (hg38), we used the RIsearch2 (v2.1) tool [39]. We allowed up to six mismatches between gRNAs and off-targets that is achieved with following settings: -s 1:20 -l 0 -m 6:0 -e 1000 –noGUseed -p3. Then, these predictions were filtered according to valid NGG, NGA, and NAG PAM sequences, and computation of all off-targeting or specificity scores for these datasets was performed as explained above.

For the off-target prediction of gRNAs, we chose the RIsearch2 program due to its high-speed performance and flexibility. It is originally proposed as an RNA–RNA interaction prediction tool that uses a seed-and-extend framework. However, by passing the parameters -s 1:20 -l 0 -m 6:0, we have only exploited its suffix array-based seed localization step, finding all off-target regions in the human (hg38) genome that have up to six mismatches with given 20-nt-long gRNA. Note that we ignore all the energies computed by RIsearch2 program and recompute the gRNA–DNA interaction energies within our pipeline.

### On-target efficiency datasets

To investigate the relationship between specificity and on-target cleavage efficiency of gRNAs, we used two different datasets, Doench2015 [40] and Wang2015 [41]. However, data for both datasets was also taken from the Haeussler benchmark study [11]. The downloaded data is already processed and includes the gRNA sequences and their cleavage efficiency measured as described in [11]. Doench2015 dataset includes 881 gRNAs with on-target modulation frequencies ranging between 0 and 1, whereas Wang2015 dataset includes 2921 gRNAs with frequencies ranging between −10 and 2. The specificity score computation of these 3802 gRNAs was performed with the same benchmark settings.

### Webserver

For the off-targeting assessment of CRISPR-Cas9 gRNAs with CRISPRoff and CRISPRspec scores, we created a webserver that meets the needs of different use cases. In the simplest use case, one can upload a gRNA sequence together with its set of predicted off-targets and the webserver returns the computed CRISPRoff scores together with the corresponding CRISPRspec specificity score of the gRNA, focusing solely on the given set of off-targets. For simplicity, the user can upload the off-target prediction set in different file formats as well, such as RIsearch2 [39] or Cas-OFFinder [47] result files. In this use case, the webserver is not limited to any organisms. Given off-targets can be based on any organism, however, for accurate CRISPRspec scorings, given off-target data must be genome-wide and must include the intended on-target sequence as well. Besides, repeated off(on)-target sites in the genome must be given separately as independent target sequences.

In case of missing off-target prediction data for gRNAs or when comparing multiple gRNA designs, the webserver performs the off-target predictions itself, using the RIsearch2 program (v2.1) in the background on a user-selected organism. In this case, the webserver outputs the CRISPRspec scores of the gRNAs under consideration together with gRNA-specific links to access the CRISPRoff scores of predicted off-target regions. In this use case, on-target and off-target sequences of all potential gRNAs can also be deployed into the UCSC browser [48] with one click for more detailed investigations. The webserver and download links for the scripts that are actively used at the back-end of the webserver are accessible through https://rth.dk/resources/crispr/.

## Additional files

Additional file 1 Supplementary document includes Supplementary **Figures S1–S6** and Supplementary **Tables S1–S3.** (PDF 882 kb)

Additional file 2 Source code of CRISPRspec and CRISPRoff. (TAR 12,511 kb)
