# Supplementary material for: CRISPR-Cas9 off-targeting assessment with nucleic acid duplex energy parameters
Source: Genome Biol. 2018 Oct 26;19:177. doi: 10.1186/s13059-018-1534-x (PMC6203265; doi:10.1186/s13059-018-1534-x)
Supplement: Supplementary file 1 — Supplementary document includes Supplementary Figures S1–S6 and Supplementary Tables S1–S3. (PDF 882 kb) [file 13059_2018_1534_MOESM1_ESM.pdf]

CRISPR-Cas9 off-targeting assessment with nucleic acid duplex energy parameters (Supplementary Document)

Ferhat Alkan, Anne Wenzel, Christian Anthon, Jakob Hull Havgaard, and Jan Gorodkin*

Center for non-coding RNA in Technology and Health, Department of Veterinary and Animal Sciences, University of Copenhagen, Grønnegårdsvej 3, 1870, Frederiksberg, Denmark

September 10, 2018

*To whom correspondence should be addressed. Tel: +45 353 33578; Fax: +45 353 34704; Email: gorodkin@rth.dk

1 Supplementary Material for Results

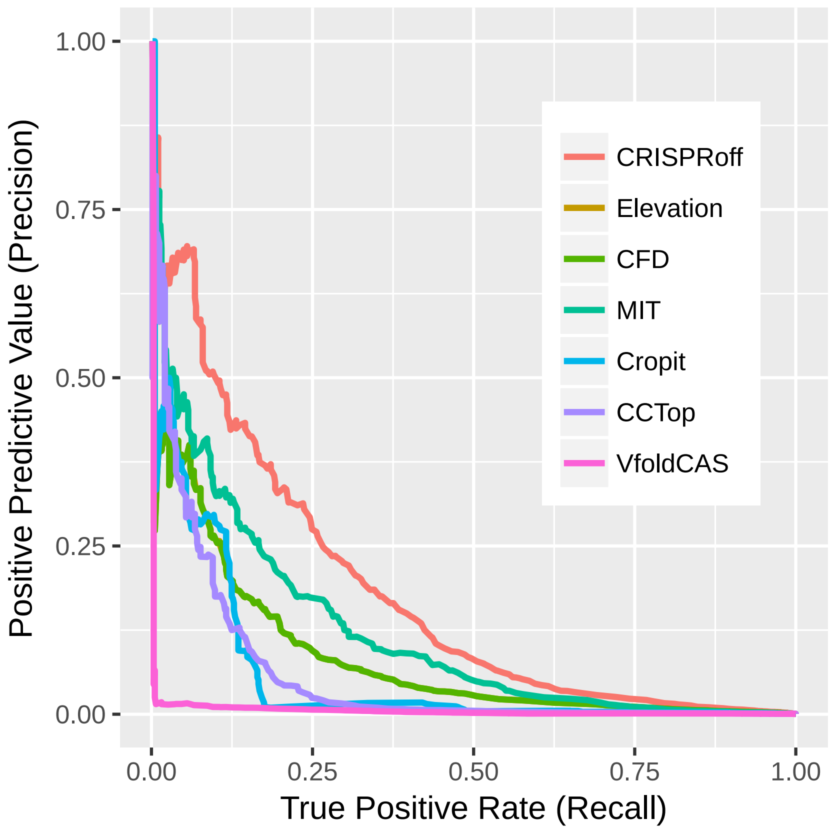

Supplementary Figure S1: Precision-recall curve analysis of off-target scoring methods when benchmarked with Haeussler dataset, allowing up to six mismatches, and NGG, NAG and NGA PAM sequences for off-targeting. PR curves for CFD and Elevation methods largely overlap and `CRISPRoff` shows the best performance with the largest area under its PR curve.

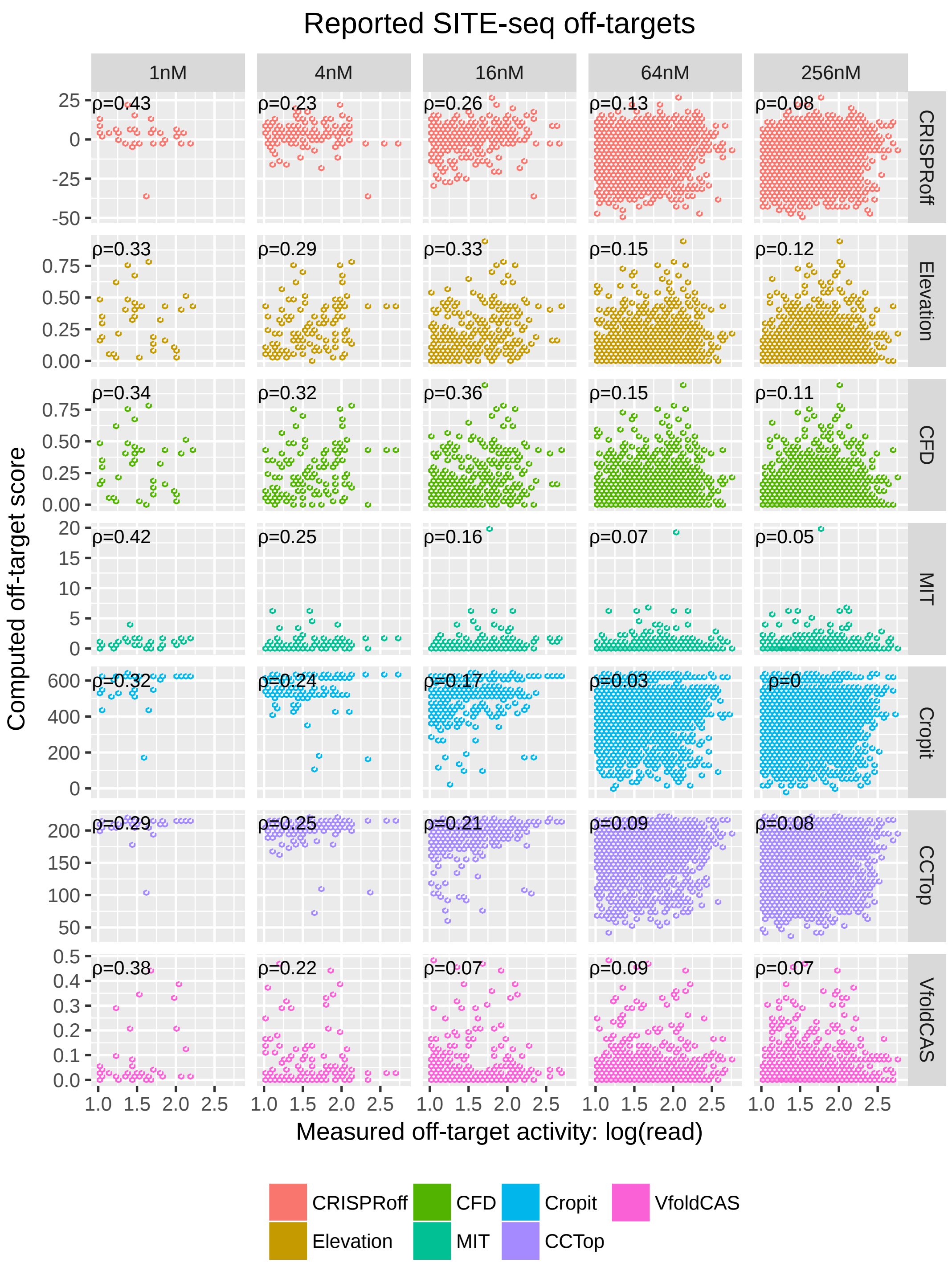

Supplementary Figure S2: Method-specific off-target score vs. measured off-target activity scatterplots (hexagonal binned) with all reported off-targets from the SITE-seq dataset at different concentrations. Measured off-target activity, given on the x-axis, corresponds to the logarithm of read counts reported for that specific off-target region. Pearson correlation coefficient between x and y axis variables are given on the top left corner of each plot.

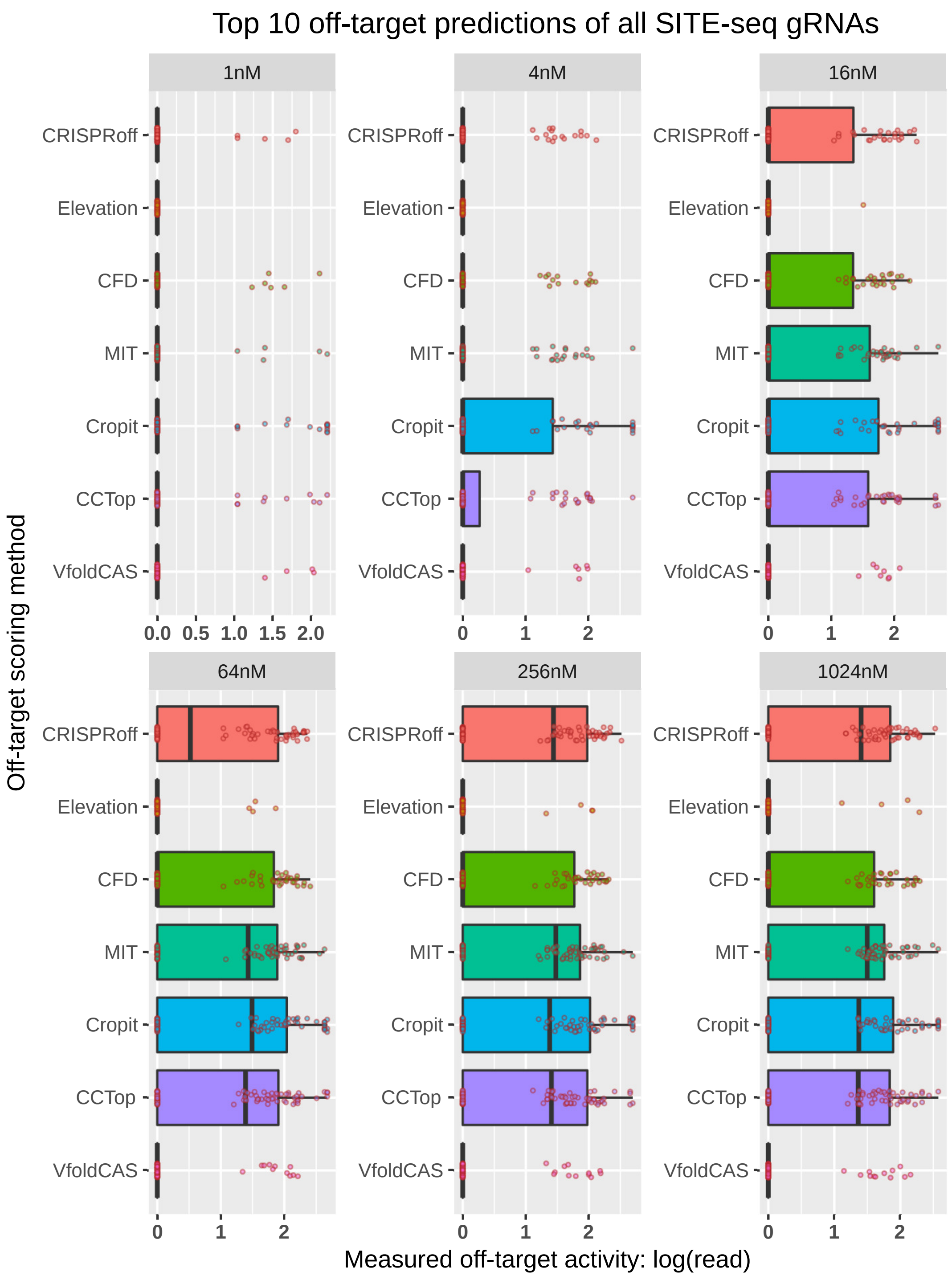

Supplementary Figure S3: SITE-seq measured (concentration-specific) off-target activity distributions of method-specific top predictions (80 in total; top 10 for all 8 experiments, each with a unique gRNA). Distributions are given separately for each method in box plot format combined with log(read) values for each off-target prediction as dot plots. Value 0 in x-axis corresponds to no experimental support for that off-target prediction.

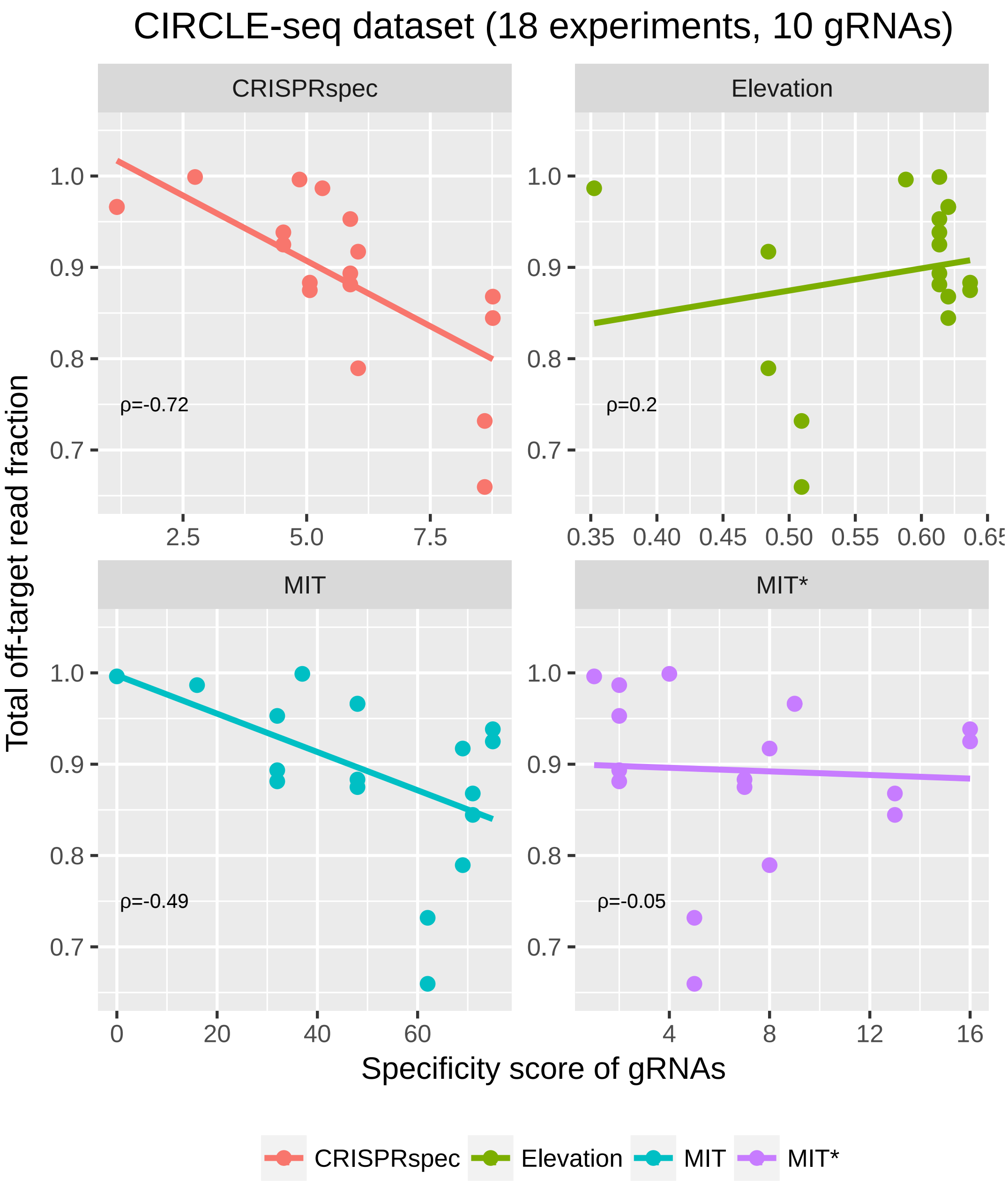

Supplementary Figure S4: Total off-target activity reported by the CIRCLE-seq experiments vs. method-specific specificity scores for 10 unique gRNAs over 18 experiments. For each gRNA, the `CRISPRspec` and MIT* scores have been computed with the same set of off-target predictions allowing up to six mismatches, whereas Elevation scores are based on its own prediction set (up to six mismatches) and MIT score has been computed with CRISPOR tool allowing up to four mismatches in off-target predictions by default. Fitted lines are shown together with the Pearson correlation coefficient between x and y axis variables in the bottom left corner of each subplot.

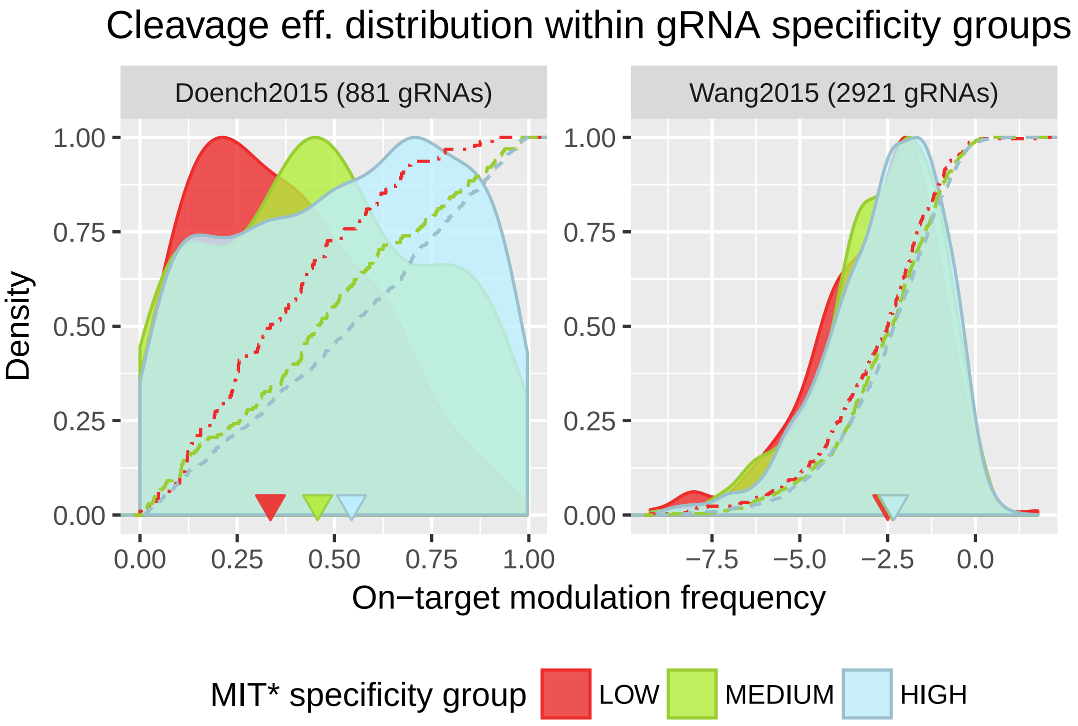

Supplementary Figure S5: On-target modulation frequency distribution of gRNAs that are binned into low, medium and high specificity groups using MIT* method. Distributions are given as kernel density estimates (filled curves) together with the cumulative distribution function (dashed lines) of on-target modulation frequencies for each specificity group, separately for each dataset. Given modulation frequencies represents the cleavage efficiency of the intended on-target and are dataset specific. Triangles on the x-axis indicate the median values.

2 Supplementary Material for Materials and Methods

2.1 Used Energy Models for Nucleic Acid Duplexes

Stability of nucleic acid duplexes are mainly dependent on Watson–Crick base pairs (A–T(U) and C–G), and consecutive base pairings are the foundation for such duplexes. To predict their stability, energy models and their thermodynamic parameters are proved to be quite useful and they have been extensively used to determine their intra- and inter-molecular structure [1]. With the help of these models, one could determine the free energy of nucleic acid duplexes which further implies their stability. The main contributor to the free energy of a duplex is usually the stacked base-pairs and they are simply computed with a nearest neighbor strategy within these models. Depending on which base pair is stacked on which pair, free energy contribution show differences. This is also the same for mismatches. Mismatches in nucleic acid duplexes create interior loops and some mismatches are more favorable than others. To be able to determine all these parameters for different nucleic acid duplexes (RNA–RNA, DNA–DNA, RNA–DNA) we make use of the models explained below to compute their free energies.

2.1.1 RNA–RNA duplex energy model

In the approximate free energy model for Cas9-target binding, we implicitly use the Turner RNA–RNA energy parameters [2] when computing ΔG_U, the free energy of gRNA intramolecular structure, with RNAfold [1]. The Turner model can also be used to determine the free energy of RNA–RNA duplexes. Even though there is no RNA–RNA duplex directly involved in the Cas9–gRNA–DNA bindings, we further use the energy parameters of RNA–RNA duplexes to determine the energy parameters for RNA–DNA duplexes, see section 2.1.3.

Nearest neighbor energy parameters of the Turner energy model are given in Supplementary Table S1. When computing the free energy of the duplex we simply sum the energy contributions of stacked base pairs and penalties for interior loops. Note that, for interior loops with 2 or 4 nucleotides, this model provides specific free energy parameters [data not shown here]. In any RNA–RNA duplex, X number of consecutive mismatches that are between two base pairs correspond to 2*X nts interior loop. To compute the free energy penalty of interior loops that are longer than 4 nt, a length-specific loop initiation penalty is simply summed with the energy contribution of the first and last matches in the loop. Note that, in this model, a G–U wobble pair is not considered as a mismatch. In the contrary, it is treated as valid base pair for all energy computations.

RNA–RNA duplex (Turner 2004)

Initiation	A(G)-U end	Internal loop size								
		2 nt	4 nt	6 nt	8 nt	10 nt	12 nt	14 nt	16 nt	18 nt
4.09	0.45	-	-	2	2.31	2.55	2.75	2.92	3.06	3.19

Nearest neighbor stacking energy parameters

$5' \rightarrow 3'$ \ $3' \rightarrow 5'$	AA	AC	AG	AU	CA	CC	CG	CU	GA	GC	GG	GU	UA	UC	UG	UU
AA	-	-	-	0.7	-	-	-	0.7	-	-	-	-0.1	0.7	0.7	-0.1	-0.93
AC	-	-	0	-	-	-	0	-	-	-	-0.8	-	0.7	0.7	-2.24	0.7
AG	-	0	-	0.7	-	0	-	0.7	-	-0.8	-	-0.1	-0.3	-2.08	-0.5	-0.55
AU	0.7	-	0.7	-	0.7	-	0.7	-	-0.1	-	-0.1	-	-1.1	0.7	-1.36	0
CA	-	-	-	0.7	-	-	-	0.7	0	0	-0.8	-2.11	-	-	-	0.7
CC	-	-	0	-	-	-	0	-	0	0	-3.26	0	-	-	0	-
CG	-	0	-	0.7	-	0	-	0.7	-1	-2.36	-1.2	-1.41	-	0	-	0.7
CU	0.7	-	0.7	-	0.7	-	0.7	-	-2.08	0	-2.11	-0.7	0.7	-	0.7	-
GA	-	-	-	-0.3	0	0	-0.8	-2.35	-	-	-	-0.5	0.7	0.7	-0.1	-1.27
GC	-	-	-1	-	0	0	-3.42	0	-	-	-1.2	-	0.7	0.7	-2.51	0.7
GG	-	-1	-	-0.3	-1	-3.26	-1.2	-1.53	-	-1.2	-	-0.5	-0.3	-2.11	-0.5	-0.5
GU	-0.3	-	-0.3	-	-2.24	0	-2.51	-0.7	-0.5	-	-0.5	-	-1.36	0.7	0.47	-0.25
UA	0.7	0.7	-0.1	-1.33	-	-	-	0	0.7	0.7	-0.1	-1	-	-	-	0
UC	0.7	0.7	-2.35	0.7	-	-	0	-	0.7	0.7	-1.53	0.7	-	-	-0.7	-
UG	-0.3	-2.11	-0.5	-1	-	0	-	0.7	-0.3	-1.41	-0.75	0.3	-	-0.7	-	0
UU	-0.93	0.7	-1.27	0	0.7	-	0.7	-	-0.55	0.7	-0.5	-0.25	0	-	0	-

Supplementary Table S1: Summary of Turner04 model nearest neighbor energy parameters for RNA–RNA duplexes. All units are given in kcal/mol.

2.1.2 DNA–DNA duplex energy model

We determine the free energy of DNA–DNA duplexes using the SantaLucia [3] and Allawi [4] energy models. Base pair stacking energies and interior loop penalties are taken from the former model whereas the energy contribution of G–T pairs comes from the latter. Nearest neighbor energy parameters of the energy model for DNA–DNA duplexes are given in Supplementary Table S2.

Within the approximate free energy computation of Cas9-target binding, we use these parameters to compute ΔG_O, the free energy penalty to open the DNA–DNA duplex at the target region. Since targeted DNA region only consists of matches, $\Delta G_O^{DNA:DNA}$ parameters constitute only the portion of the values in the table below. We make use of the other parameters to determine the energy parameters for RNA–DNA duplexes. To compute the free energy penalty of interior loops, length-specific loop initiation penalty is simply summed with the energy contribution of the first and last Watson–Crick pair in the loop.

DNA–DNA duplex (SantaLucia & Hicks 2004)

Initiation	A-T end	Internal loop size								
		2 nt	4 nt	6 nt	8 nt	10 nt	12 nt	14 nt	16 nt	18 nt
1.96	0.05	0	3.6	4.4	4.8	4.9	5.2	5.4	5.6	5.8

Nearest neighbor stacking energy parameters

		$3' \rightarrow 5'$															
		AA	AC	AG	AT	CA	CC	CG	CT	GA	GC	GG	GT	TA	TC	TG	TT
$5' \rightarrow 3'$	AA	-	-	-	0.69	-	-	-	1.33	-	-	-	0.74	0.61	0.88	0.14	-1
	AC	-	-	0.17	-	-	-	0.47	-	-	-	-0.52	-	0.77	1.33	-1.44	0.64
	AG	-	0.43	-	0.69	-	0.79	-	1.33	-	0.11	-	0.74	0.02	-1.28	-0.13	0.71
	AT	0.61	-	0.69	-	0.77	-	1.33	-	0.02	-	0.74	-	-0.88	0.73	0.07	0.69
	CA	-	-	-	0.92	-	-	-	1.05	0.43	0.75	0.03	-1.45	-	-	-	0.75
	CC	-	-	0.81	-	-	-	0.79	-	0.79	0.7	-1.84	0.62	-	-	0.98	-
	CG	-	0.75	-	0.92	-	0.7	-	1.33	0.11	-2.17	-0.11	-0.47	-	0.4	-	0.98
	CT	0.88	-	0.92	-	1.33	-	1.33	-	-1.28	0.4	-0.32	-0.12	0.73	-	0.98	-
	GA	-	-	-	0.42	0.17	0.81	-0.25	-1.3	-	-	-	0.44	1.15	1.15	1.15	0.34
	GC	-	-	-0.25	-	0.47	0.79	-2.24	0.62	-	-	-1.11	-	1.33	1.33	-0.59	1.15
	GG	-	0.03	-	0.42	-0.52	-1.84	-1.11	0.08	-	-0.11	-	0.44	1.15	-0.32	1.15	0.74
	GT	0.14	-	0.42	-	-1.44	0.98	-0.59	0.45	-0.13	-	0.44	-	0.07	1.15	1.15	1.15
	TA	0.69	0.92	0.42	-0.58	-	-	-	0.97	0.74	0.92	0.74	0.43	-	-	-	0.68
	TC	1.33	1.05	-1.3	0.97	-	-	0.62	-	1.33	1.33	0.08	0.97	-	-	0.45	-
	TG	0.74	-1.45	0.44	0.43	-	0.62	-	0.97	0.74	-0.47	0.74	0.52	-	-0.12	-	0.69
	TT	-1	0.75	0.34	0.68	0.64	-	0.97	-	0.71	0.98	0.74	0.69	0.69	-	0.69	-

Supplementary Table S2: Nearest neighbor energy parameters for DNA–DNA duplexes. All units are given in kcal/mol.

2.1.3 RNA–DNA duplex energy model

In comparison to RNA–RNA and DNA–DNA duplexes, thermodynamic stability of RNA–DNA duplexes has been poorly investigated. For the free energy computation of these duplexes, the Sugimoto energy model [5, 6] provides the nearest neighbor energy parameters of Watson–Crick base pairings, however, parameters for internal loops are still not known apart from some single mismatches from Watkins [7]. To be able to compute ΔG_B, the free energy of the gRNA–DNA duplex, we used a simple averaging approach of RNA–RNA and DNA–DNA parameters to complete the missing parameters of the Sugimoto–Watkins combined model. We present the parameters from Sugimoto–Watkins combined model in Supplementary Table S3 together with the missing parameters generated here. Note that we consider all G–T and U–G pairs as mismatches in the RNA–DNA duplex energy model.

In summary, our final RNA:DNA duplex energy model has the following components: stacking, internal loops, and external loops. Computation of $\Delta G^{RNA:DNA}_{g[i,i+1]:t[i,i+1]}$ differs depending on the component that it is part of.

<u>**Stacking:**</u> When there are Watson–Crick base pairs between the RNA and DNA at position i and $i+1$, it is considered as part of stacking:

$$\Delta G^{RNA:DNA}_{g[i,i+1]:t[i,i+1]} = \Delta G^{RNA:DNA}_{g[i,i+1]:t[i,i+1]\text{stacking}} \tag{1}$$

The value of $\Delta G^{RNA:DNA}_{g[i,i+1]:t[i,i+1]\text{stacking}}$ is directly read from Supplementary Table S3.

<u>**Internal loop:**</u> An internal loop is a stretch of mismatches enclosed by two Watson–Crick base pairs. In our model, there are two types of internal loops (as shown in Supplementary Equation 2): Short internal loops (2 or 4 nt, formed by 1 or 2 mismatches), or long internal loops. For short loops (2 or 4 nt), the RNA:RNA Turner model has special energies dependent on the nucleotides. Furthermore G–U base pairs are treated as matches in the RNA:RNA model whereas they are considered as mismatches in the DNA:DNA model. In our RNA:DNA model G–U base pairs are treated as mismatches, however, the energy computation takes into account that they are possible to stack on the neighboring base pairs for the RNA:RNA model. The equations for computing the positional contributions in internal loops (IL) are:

$$\Delta G^{RNA:DNA}_{g[k,k+1]:t[k,k+1]} = \begin{cases} \frac{\Delta G^{RNA:DNA}_{g[i,j]:t[i,j]IL_{short}}}{(j-i)} & \text{if } (j-i) \leq 3 \\ \frac{\Delta G^{RNA:DNA}_{g[i,j]:t[i,j]IL_{long}}}{(j-i)} & \text{otherwise} \end{cases} \tag{2}$$

where $i \leq k < j$, and only position i and j are base-paired.

Below, we provide the equations for different sub-cases of internal loops.

For short internal loops where $j = i+2$ or $j = i+3$,

$$\Delta G^{RNA:DNA}_{g[i,j]:t[i,j]IL_{short}} = \frac{\Delta G^{RNA:RNA}_{g[i,j]:t[i,j]IL} + \Delta G^{DNA:DNA}_{g[i,j]:t[i,j]IL}}{2} \tag{3}$$

The calculation of $\Delta G^{RNA:RNA}_{g[i,j]:t[i,j]IL}$ is explained below, and the energy of short DNA–DNA internal loop is computed as follows:

$$\Delta G^{DNA:DNA}_{g[i,j]:t[i,j]IL} = \Delta G^{DNA:DNA}_{g[i,i+1]:t[i,i+1]\text{stacking}} + \Delta G^{DNA:DNA}_{g[j-1,j]:t[j-1,j]\text{stacking}} + \Delta G^{DNA:DNA}_{\text{loop penalty, }[2\times(j-i-1)]nt} \tag{4}$$

$\Delta G^{DNA:DNA}_{\text{loop penalty, }[2\times(j-i-1)]nt}$ is a length dependent energy, which can be found together with the

stacking energies in Supplementary Table S2. Note that $[2\times(j-i-1)]nt$ refers to the internal loop size and size-specific loop penalties are given on the top panel of this table. On the other hand, the energy of short RNA–RNA internal loops are computed slightly differently due to the stacking of G-U pairs.

When $j = i + 2$,

$$\Delta G^{RNA:RNA}_{g[i,j]:t[i,j]IL} = \begin{cases} \Delta G^{RNA:RNA}_{g[i,j]:t[i,j]IL_{short}} & i+1 \text{ is not a G–U pair} \\ \sum_k^{i\leq k<j} \Delta G^{RNA:RNA}_{g[k,k+1]:t[k,k+1]\text{stacking}} & i+1 \text{ is a G–U pair} \end{cases} \quad (5)$$

In the first case, the unpaired nucleotides of the loop is not a G–U pair (or U–G), and in the second they are. The pairs opening and closing the loop are always not G–U pairs. The $\Delta G^{RNA:RNA}_{g[i,j]:t[i,j]IL_{short}}$ is the loop energy which depends on the length and the nucleotides involved in the loop, and Turner model provides special energies for such cases. The full set of parameters for the RNA:DNA short internal loops are available through download of the source code. However, the value of $\Delta G^{RNA:RNA}_{g[k,k+1]:t[k,k+1]\text{stacking}}$ is directly read from Supplementary Table S1.

When $j = i + 3$,

$$\Delta G^{RNA:RNA}_{g[i,j]:t[i,j]IL} = \begin{cases} \Delta G^{RNA:RNA}_{g[i,j]:t[i,j]IL_{short}} & \text{no G–U pairs} \\ \Delta G^{RNA:RNA}_{g[i,i+1]:t[i,i+1]\text{stacking}} + \Delta G^{RNA:RNA}_{g[i+1,i+3]:t[i+1,i+3]IL_{short}} & i+1 \text{ is a G–U pair} \\ \Delta G^{RNA:RNA}_{g[i,i+2]:t[i,i+2]IL_{short}} + \Delta G^{RNA:RNA}_{g[i+2,i+3]:t[i+2,i+3]\text{stacking}} & i+2 \text{ is a G–U pair} \\ \sum_k^{i\leq k<j} \Delta G^{RNA:RNA}_{g[k,k+1]:t[k,k+1]\text{stacking}} & \text{both G–U pairs} \end{cases} \quad (6)$$

When $j > i + 3$,

Within the RNA:DNA energy model, the energy of long internal loops (second part of equation (2)) are computed using:

$$\Delta G^{RNA:DNA}_{g[i,j]:t[i,j]IL_{long}} = \Delta G^{RNA:DNA}_{g[i,i+1]:t[i,i+1]\text{stacking}} + \Delta G^{RNA:DNA}_{g[j-1,j]:t[j-1,j]\text{stacking}} + \Delta G^{RNA:DNA}_{\text{loop penalty, }[2\times(j-i-1)]nt} \quad (7)$$

$\Delta G^{RNA:DNA}_{\text{loop penalty, }[2\times(j-i-1)]nt}$ is length-dependent energy penalty for interior loops which can be found together with the stacking energies in Supplementary Table S3.

External loop: When position i is part of an external loop, that is a stretch of mismatches in the beginning or end of the interaction and therefore only enclosed by one Watson–Crick base pair rather than two pairs, the energy contribution of position i is equal to 0 kcal/mol. However, if there is an external loop in the interaction, an extra energy contribution is introduced for the first (or last) Watson–Crick base pair closing the loop, when it is an A–T base pair [2, 3]. The A–T closing energy is calculated using:

$$\Delta G^{RNA:DNA}_{g[i]:t[i]\text{A–T closing}} = \left(\Delta G^{RNA:RNA}_{g[i]:t[i]\text{A–T closing}} + \Delta G^{DNA:DNA}_{g[i]:t[i]\text{A–T closing}}\right)/2 \quad (8)$$

Using parameters from Supplementary Table S1 and S2. The precalculated energy (0.25 kcal/mol) has been added to Supplementary Table S3. If the external loop is in the beginning of the interaction, here at position i, the energy contribution for $\Delta G^{RNA:DNA}_{g[i,i+1]:t[i,i+1]}$ is replaced by:

$$\Delta G^{RNA:DNA}_{g[i,i+1]:t[i,i+1]} = \Delta G^{RNA:DNA}_{g[i,i+1]:t[i,i+1]} + \Delta G^{RNA:DNA}_{g[i]:t[i]\text{A–T closing}} \quad (9)$$

Similarly if there is an external loop at the end of the interaction, here closed by a base pair at $i + 1$:

$$\Delta G^{RNA:DNA}_{g[i,i+1]:t[i,i+1]} = \Delta G^{RNA:DNA}_{g[i,i+1]:t[i,i+1]} + \Delta G^{RNA:DNA}_{g[i+1]:t[i+1]\text{A–T closing}} \quad (10)$$

RNA–DNA duplex

Initiation	A-T(U) end	Internal loop size								
		2 nt	4 nt	6 nt	8 nt	10 nt	12 nt	14 nt	16 nt	18 nt
3.1	0.25	-	-	3.2	3.555	3.725	3.975	4.16	4.33	4.495

Nearest neighbor stacking energy parameters

RNA $5' \to 3'$	DNA $3' \to 5'$															
	AA	AC	AG	AT	CA	CC	CG	CT	GA	GC	GG	GT	TA	TC	TG	TT
AA	-	-	-	1.36	-	-	-	1.015	-	-	-	0.32	1.07	0.79	0.02	-1
AC	-	-	0.19	-	-	-	0.235	-	-	-	-0.66	-	0.735	1.64	-2.1	0.67
AG	-	0.21	-	0.695	-	0.395	-	1.015	-	-0.345	-	0.32	-0.14	-1.8	0.31	0.08
AU	1.85	-	0.695	-	0.735	-	1.015	-	-0.04	-	0.32	-	-0.9	0.715	-0.645	0.63
CA	-	-	-	0.81	-	-	-	1.7	0.9	0.375	-0.385	-0.9	-	-	-	0.725
CC	-	-	0.405	-	-	-	0.73	-	0.395	1.04	-2.1	0.31	-	-	0.49	-
CG	-	0.375	-	0.81	-	0.46	-	1.015	-0.445	-1.7	0.14	-0.94	-	0.2	-	0.84
CU	0.79	-	0.81	-	1.88	-	1.015	-	-0.9	0.2	-1.215	0.49	0.715	-	0.84	-
GA	-	-	-	0.06	0.51	0.405	-0.525	-1.3	-	-	-	0.5	0.925	0.925	0.525	-0.465
GC	-	-	-0.625	-	0.235	0.96	-2.7	0.31	-	-	-0.83	-	1.015	1.015	-1.55	0.925
GG	-	-0.485	-	0.06	-0.76	-2.9	-0.58	-0.725	-	-0.33	-	-0.03	0.425	-1.215	0.325	0.12
GU	-0.08	-	0.06	-	-1.1	0.49	-1.55	0.18	0.97	-	-0.03	-	-0.645	0.925	0.81	0.45
UA	1.13	0.81	0.16	-0.6	-	-	-	0.485	0.72	0.81	0.32	-0.285	-	-	-	1.21
UC	1.015	1.15	-1.5	0.835	-	-	0.31	-	1.015	1.015	-0.725	0.835	-	-	-0.02	-
UG	0.22	-1.6	0.44	-0.285	-	0.31	-	0.835	0.22	-0.94	-0.005	0.41	-	0.14	-	0.345
UU	-0.2	0.725	-0.465	1.07	0.67	-	0.835	-	0.08	0.84	0.12	0.22	1.03	-	0.345	-

Supplementary Table S3: Estimated nearest neighbor energy parameters for RNA–DNA duplexes. Parameters from Sugimoto [5, 6] and Watkins [7] models are highlighted with yellow. All units are given in kcal/mol. Note that internal loops with size 2 and 4 nt have specific free energy contributions which are not included in this table.

In Supplementary Figure S6, we provide an example gRNA–DNA interaction for which we show the details of how to compute the free energy contributions $\Delta G^{RNA:DNA}_{g[i,i+1]:t[i,i+1]}$, $1 \leq i \leq 19$. Note that, we focus on the gRNA sequence in 5' to 3' order, whereas the direction for the DNA sequence is from 3' to 5'. For ease of explanation, the interaction is divided into 6 parts (A–F) and within each part different positional energies are computed.

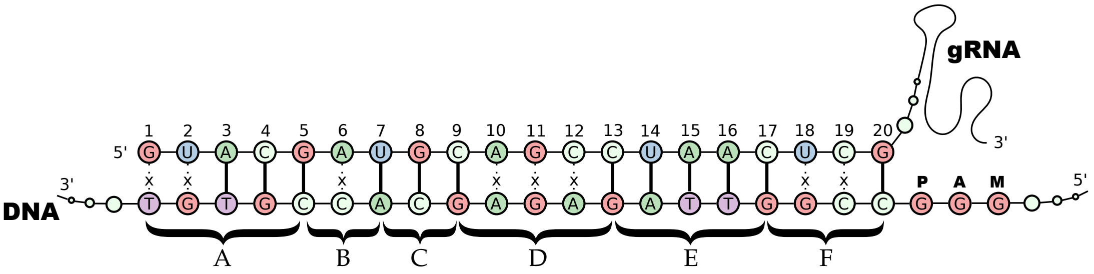

Supplementary Figure S6: An example showing a gRNA–DNA (g–t) interaction where free energy contributions are computed specifically for every pair of positions ($\Delta G^{RNA:DNA}_{g[i,i+1]:t[i,i+1]}$, $1 \leq i \leq 19$). For ease of explanation, we divide the interaction into six different parts (A–F). Part B, D and F of the interaction are considered as interior loops. Part C and E are examples of stacked base pairs and part A shows an external loop where G–T and U–G base pairs are treated as mismatches.

Part A: In this part of the interaction we compute the positional free energy contributions for positions $1 \leq i \leq 4$. While the stacking of the nucleotides at positions 4 and 5 are not part of the end stacking it is included here to shorten the example. As G–T and U–G base pairs are not allowed in the RNA:DNA model positions 1 and 2 forms an external loop which is closed by the base pair at position 3. So the $\Delta G^{RNA:DNA}_{g[1,2]:t[1,2]}$ and $\Delta G^{RNA:DNA}_{g[2,3]:t[2,3]}$ positional energy contributions are equal to 0 kcal/mol. A–T, C–G and G–C pairs are valid base pairs and according to Supplementary Table S3; using Supplementary Equation (1) and (9): $\Delta G^{RNA:DNA}_{g[3,4]:t[3,4]} = \Delta G^{RNA:DNA}_{AC:TG \text{ stacking}} + \Delta G_{g[3]:t[3]\text{A–T closing}} = -2.1\,\text{kcal/mol} + 0.25\,\text{kcal/mol} = -1.85\,\text{kcal/mol}$, and Supplementary Equation (1): $\Delta G^{RNA:DNA}_{g[4,5]:t[4,5]} = \Delta G^{RNA:DNA}_{CG:GC \text{ stacking}} = -1.7\,\text{kcal/mol}$.

Part B: This is an interior loop with 2 nt which is a short internal loop. According to the Turner RNA–RNA energy model, the free energy of this interior loop is $\Delta G^{RNA:RNA}_{g[5,7]:t[5,7]\ IL_{short}} = \Delta G^{RNA:RNA}_{GAU:CAA\ IL_{short}} = 1.2\,\text{kcal/mol}$ (Supplementary Equation (5) (not a G-U pair), parameters not shown) and the DNA–DNA part becomes $\Delta G^{DNA:DNA}_{g[5,7]:t[5,7]\ IL} = \Delta G^{DNA:DNA}_{GA,CC \text{ stacking}} + \Delta G^{DNA:DNA}_{AT,AC \text{ stacking}} + \Delta G^{DNA:DNA}_{\text{loop penalty, } 2nt} = 0.81 + 0.77 + 0 = 1.58\,\text{kcal/mol}$, according to Supplementary Equation (4) and Supplementary Table S2. Lastly, Supplementary Equations (2) and (3) becomes: $\Delta G^{RNA:DNA}_{g[5,6]:t[5,6]} = \Delta G^{RNA:DNA}_{g[6,7]:t[6,7]} = ((1.2 + 1.58)/2)/2{=}0.695\,\text{kcal/mol}$. Note that averaged energy is distributed equally to every position (2 in this case) forming the loop according to Supplementary Equation (2).

Part C: Free energy contribution of stackings (Supplementary Equation (1)) can be read from Supplementary Table S3.
$\Delta G^{RNA:DNA}_{g[7,8]:t[7,8]} = \Delta G^{RNA:DNA}_{UG:AC \text{ stacking}} = -1.6\,\text{kcal/mol}$ and
$\Delta G^{RNA:DNA}_{g[8,9]:t[8,9]} = \Delta G^{RNA:DNA}_{GC:CG \text{ stacking}} = -2.7\,\text{kcal/mol}$.

Part D: This is an interior loop with 6 nt. As it is the case for all interior loops longer than 4 nt, we sum the length-specific loop penalty (Internal loop size: $6nt$) with the contribution of loop-closing matches, Supplementary Equations (2) and (7).
$\Delta G^{RNA:DNA}_{g[9,10]:t[9,10]} = \Delta G^{RNA:DNA}_{g[10,11]:t[10,11]} = \Delta G^{RNA:DNA}_{g[11,12]:t[11,12]} = \Delta G^{RNA:DNA}_{g[12,13]:t[12,13]} =$
$(\Delta G^{RNA:DNA}_{CA:GA \text{ stacking}} + \Delta G^{RNA:DNA}_{CC:AG \text{ stacking}} + \Delta G^{RNA:DNA}_{\text{loop penalty, } 6nt})/4 =$
$(0.9\,\text{kcal/mol} + 0.405\,\text{kcal/mol} + 3.2\,\text{kcal/mol})/4 = 1.126\,\text{kcal/mol}$

Part E: Free energy contribution of matches can be read from Supplementary Table S3 (Supplementary Equation (1)).
$\Delta G^{RNA:DNA}_{g[13,14]:t[13,14]} = \Delta G^{RNA:DNA}_{CU:GA \text{ stacking}} = -0.9\,\text{kcal/mol}$,
$\Delta G^{RNA:DNA}_{g[14,15]:t[14,15]} = \Delta G^{RNA:DNA}_{UA:AT \text{ stacking}} = -0.6\,\text{kcal/mol}$,

$\Delta G^{RNA:DNA}_{g[15,16]:t[15,16]} = \Delta G^{RNA:DNA}_{AA:TT \text{ stacking}} = -1.0\,\text{kcal/mol}$ and
$\Delta G^{RNA:DNA}_{g[16,17]:t[16,17]} = \Delta G^{RNA:DNA}_{AC:TG \text{ stacking}} = -2.1\,\text{kcal/mol}$.

Part F: This is an interior loop with 4 nt. Using Supplementary Equation (3) ΔG is split into the RNA and DNA parts. The RNA part is calculated with Supplementary Equation (6). Since position 18 is a U–G pair the *"i + 1 is a G–U pair"* part of the equation is used: $\Delta G^{RNA:RNA}_{g[17,20]:t[17,20]IL} = \Delta G^{RNA:RNA}_{g[17,18]:t[17,18]\text{stacking}} + \Delta G^{RNA:RNA}_{g[18,20]:t[18,20]IL_{short}} = -2.11\,\text{kcal/mol} + 1.7\,\text{kcal/mol} = 0.41\,\text{kcal/mol}$. The stacking energy can be found in Supplementary Table S1, but the loop energy is not shown here, it is however available in the source code. For the DNA part Supplementary Equation (4) becomes: $\Delta G^{DNA:DNA}_{g[17:20]:t[17:20]IL} = \Delta G^{DNA:DNA}_{CT,GG \text{ stacking}} + \Delta G^{DNA:DNA}_{CG,CC \text{ stacking}} + \Delta G^{DNA:DNA}_{\text{loop penalty, } 4nt} = -0.32 + 0.7 + 3.6 = 3.98\,\text{kcal/mol}$, where parameters are from Supplementary Table S2. Finally using Supplementary Equations (2) and (3): $\Delta G^{RNA:DNA}_{g[17,18]:t[17,18]} = \Delta G^{RNA:DNA}_{g[18,19]:t[18,19]} = \Delta G^{RNA:DNA}_{g[19,20]:t[19,20]} = ((-0.41+3.98)/2)/3 = 0.595\,\text{kcal/mol}$.

References

[1] Lorenz, R., Bernhart, S.H., Honer Zu Siederdissen, C., Tafer, H., Flamm, C., Stadler, P.F., Hofacker, I.L.: ViennaRNA Package 2.0. Algorithms Mol Biol **6**, 26 (2011)

[2] Turner, D.H., Mathews, D.H.: NNDB: the nearest neighbor parameter database for predicting stability of nucleic acid secondary structure. Nucleic Acids Res. **38**(Database issue), 280–282 (2010)

[3] SantaLucia, J., Hicks, D.: The thermodynamics of DNA structural motifs. Annu Rev Biophys Biomol Struct **33**, 415–440 (2004)

[4] Allawi, H.T., SantaLucia, J.: Thermodynamics and NMR of internal G.T mismatches in DNA. Biochemistry **36**(34), 10581–10594 (1997)

[5] Sugimoto, N., Nakano, S., Katoh, M., Matsumura, A., Nakamuta, H., Ohmichi, T., Yoneyama, M., Sasaki, M.: Thermodynamic parameters to predict stability of RNA/DNA hybrid duplexes. Biochemistry **34**(35), 11211–11216 (1995)

[6] Sugimoto, N., Nakano, M., Nakano, S.: Thermodynamics-structure relationship of single mismatches in RNA/DNA duplexes. Biochemistry **39**(37), 11270–11281 (2000)

[7] Watkins, N.E., Kennelly, W.J., Tsay, M.J., Tuin, A., Swenson, L., Lee, H.R., Morosyuk, S., Hicks, D.A., Santalucia, J.: Thermodynamic contributions of single internal rAdA, rCdC, rGdG and rUdT mismatches in RNA/DNA duplexes. Nucleic Acids Res. **39**(5), 1894–1902 (2011)
